# Familicide in Canada, 2010 to 2019

**DOI:** 10.1177/10887679221097626

**Published:** 2022-07-06

**Authors:** Ciara Boyd, Danielle Sutton, Myrna Dawson, Angelika Zecha, Julie Poon, Anna-Lee Straatman, Peter Jaffe

**Affiliations:** 1 University of Guelph, Guelph, Canada; 2 Western University, London, Canada

**Keywords:** familicide, violence, gender, mass homicide, family homicide, domestic homicide

## Abstract

Familicide is rare; however, the high victim counts in each incident and context surrounding these killings underscore the need for further research. The purpose of this study is to gain a better understanding of the circumstances surrounding familicide in Canada. Using univariate statistics, this study analyzed 26 incidents of familicide that occurred in Canada between 2010 and 2019. The results show that familicide is a gendered crime involving primarily male accused who often target female victims, have a history of domestic violence, and commit the killings using firearms. This research highlights the importance of developing risk assessment, risk management, and safety planning strategies to address warning signs and prevent future familicides.

## Introduction

In Canada, a woman is killed by an intimate partner about every 6 days (Dawson et al., 2021). Intimate partner homicide (IPH), sometimes referred to as domestic homicide (DH), or intimate partner femicide (IPF) to capture its gender-specific elements, has received considerable research attention worldwide (Gill, 2012; Karlsson, Malén, et al., 2021). Familicide, also a gendered crime, has received minimal attention in comparison, particularly in Canada. Research demonstrates that familicide is almost exclusively perpetrated by males and typically involves the killing of two or more family members, often a current or former female partner and at least one biological child or stepchild (Leveillee et al., 2007; Mailloux, 2014; Wilson et al., 1995). The lack of research on familicide may be explained by its relative rarity (Karlsson, Antfolk, et al., 2021). For example, Wilson et al. (1995) found that only 61 incidents of familicide, involving 161 victims, occurred in Canada between 1974 and 1990. Familicide victims, thus, accounted for less than 2% of all homicide victims in Canada during that time (Wilson et al., 1995). This figure is slightly higher than in England and Wales, where familicide victims accounted for less than 1% of homicide victims (Wilson et al., 1995). Despite its rarity, such incidents are devastating for individuals left behind, the communities in which they occur, and for society, making it crucial to examine common characteristics to better understand risk and identify prevention practices.

To better understand familicide, this study examines the following research question: What do cases of familicide look like in Canada? This question explores whether familicide in Canada differs from what the literature shows regarding familicide in other countries and, if so, whether Canadian-specific prevention measures are necessary. It also highlights warning signs that may be used to develop risk assessment strategies to prevent future occurrences. Drawing from feminist and masculinities theoretical frameworks, we use data collected from the Canadian Domestic Homicide Prevention Initiative with Vulnerable Populations (CDHPIVP), a 5-year project funded by the Social Sciences and Humanities Research Council (SSHRC) that explored DH in Canada. Before turning to our study, we review prior research that has examined the phenomenon which informs our study and from which our hypotheses were derived.

## Literature Review

Familicide typically involves a male who kills multiple family members (Karlsson, Antfolk, et al., 2021). Familicide may be defined broadly, such as one family member killing multiple other family members (Fegadel & Heide, 2017; Malmquist, 1980), or narrowly, such as an individual who kills a current or former intimate partner and at least one biological child or stepchild (Liem et al., 2013; Wilson et al., 1995). The latter definition is common in familicide research (Leveillee et al., 2009; Liem & Koenraadt, 2008; Websdale, 2010) and, thus, was employed in our study. The next section summarizes the limited information known about familicide perpetrators, victims, and incident characteristics, as well as motivational factors.

### Victim and Perpetrator Characteristics

#### Perpetrator characteristics

Research shows that familicide is a male-perpetrated crime with as many as 95% of perpetrators being male (Gill, 2012; Leveillee et al., 2007; Mailloux, 2014). Most perpetrators were approximately 30- to 40-years old (Karlsson, Antfolk, et al., 2021; Liem & Koenraadt, 2008; Wilson et al., 1995) and many had a history of domestic violence (DV) perpetration, including physical, sexual, or emotional violence toward their victim(s) and/or previous partners (Dobash et al., 2007; Johnson & Sachmann, 2014; Taylor, 2018). To illustrate, Johnson and Sachmann (2014) found that 89% of familicide perpetrators had a documented history of violence, intimidation, and/or threatening acts against their partner. Similarly, Dobash et al. (2007) found that 57% of perpetrators had engaged in DV in past relationships. These figures likely underestimate the prevalence of DV as it is not always officially documented or shared informally (Auchter, 2010).

Some research has found that perpetrators of familicide suffered from mental illness (Karlsson, Malén, et al., 2021) whereas others found a history of mental illness was rare (Vinas-Racionero et al., 2017). These contradictory findings could be a product of variations in how studies define mental illness (Vinas-Racionero et al., 2017). Some define it as depression (Hamilton et al., 2013) whereas others define it as a psychotic disorder, mood disorder, or personality disorder (Liem & Koenraadt, 2008). The way researchers determine the presence of mental illness also varies, with some relying on psychiatric and psychological reports (Vinas-Racionero et al., 2017), some relying on statements from friends, family, co-workers, and law enforcement personnel (Taylor, 2018), and others not disclosing how it is determined (Aho et al., 2017). Further, some men who suffer from mental illness may be in denial or avoid seeking help, resulting in fewer reported instances (Oliver & Jaffe, 2021).

#### Victim characteristics

Given familicides are mostly male-perpetrated, they typically involve the killing of adult female partners (Karlsson, Malén, et al., 2021). In fact, research shows that between 87% and 100% of adult victims were women (Liem et al., 2013). When children were killed, gender did not appear to affect risk of victimization (Wilson et al., 1995). Adult victims were generally between the ages of 35 and 39 and child victims were often between the ages of 7 and 12 years (Karlsson, Malén, et al., 2021).

Victim-perpetrator relationship is crucial to familicide research because relationship state (i.e., whether the victim and perpetrator were in a current or estranged relationship) could increase the likelihood of familicide. Research shows that actual or threatened estrangement heightened the risk of familicide because, when a woman left the relationship, the perpetrator felt his control had been threatened or was lost (Mailloux, 2014). Familicide also appears to occur more often in legal marriages than in common-law relationships (Karlsson, Malén, et al., 2021; Mailloux, 2014).

### Incident Characteristics

According to prior literature, familicide occurs more often in rural rather than urban areas (Duwe, 2004; Gallup-Black, 2005). Familicides are often committed in locations known to the perpetrator, such as their home, often shared with their victims (Capellan & Gomez, 2018). Research also shows that most familicide perpetrators either attempt or die by suicide following the killings (Liem & Koenraadt, 2008; Taylor, 2018). For example, Liem and Reichelmann (2014) found that over half (57%) of the familicide perpetrators in their sample died by suicide.

In Canada and abroad, most familicides were committed using firearms (Auchter, 2010; Fegadel & Heide, 2017; Karlsson, Antfolk, et al., 2021). A history of DV was a risk factor for familicide, one that was heightened when weapons were previously involved in DV incidents (Stith & Amanor-Boadu, 2010). In some cases, perpetrators used diverse weapons, such as stabbing and/or beating with an object (Fegadel & Heide, 2017). The cause of death in familicides may vary depending on the geographic location of the familicide, however. Taylor (2018) suggested that perpetrators were more likely to use strangulation or stabbing as causes of death in countries where gun ownership was more restricted, such as Canada. Literature has not examined why firearms are predominantly involved in cases of familicide.

### Motivations for Familicide

A perpetrator’s motivation to commit familicide is not always clear, especially considering the large proportion that die by suicide (Mailloux, 2014). However, research suggests that motivations are commonly connected to an intimate partner (Karlsson, Malén, et al., 2021), often seen as the primary victim. To illustrate, the most documented familicidal motivations were triggered by an intimate partner leaving or threatening to leave the relationship (Duwe, 2004; Liem & Reichelmann, 2014). In such cases, the perpetrator often committed the familicide as a form of revenge and children were killed because he wanted to further harm the mother or because he saw them as an extension of her (Jaffe et al., 2014; Liem & Reichelmann, 2014).

Altruistic killings are motivated by the desire to relieve real or imagined suffering of an individual(s) (Friedman et al., 2005). This has been referred to as altruistic suicide, where a suicidal individual kills his family to protect them from living in a world without him (Wallace, 1986). In such cases, perpetrators may believe familicide is the only way of protecting their families (Duwe, 2004). For example, a husband who lost his job may fear his family would be unable to support themselves and commit the familicide because he believed they were better off dead than struggling financially. There is disagreement in the literature regarding familicides that are said to be motivated by altruism, however. For example, research suggests that cases where perpetrators were suicidal and extended the suicide onto their family were not representative of a man trying to protect his family, but rather of suicidal tendencies and an inability to see his partner/family as separate from himself (Palmero, 1994). Therefore, altruistic killings may occur because the perpetrator feels entitled to control his family.

Mental illness is represented in some cases as a motivation for familicide, although results are inconclusive for some of the reasons discussed above. For example, Aho et al. (2017) argued that perpetrators who showed signs of mental illness committed familicide because of their illness whereas Taylor (2018) argued that the likelihood of mental illness as a motive was uncommon and overrepresented in the literature.

## Hypotheses

Drawing from existing literature, we hypothesize the following in our sample:

H1: Histories of DV will be common among familicide perpetrators
H2: Many familicides will be motivated by triggering events connected to the perpetrator’s intimate partner, such as separation/estrangement
H3: Firearms will be the predominant weapon used to commit familicide
H4: The majority of familicide perpetrators will die by suicide
H5: Most familicides will occur in rural areas

Drawing from prior research, other hypotheses could be made; however, the above were chosen as they capture the characteristics of familicide with the most consensus in the literature.

## Theoretical Framework

Based on prior literature, most familicide perpetrators are males who target female primary victims (Wilson et al., 1995), which highlights the gendered nature of familicides and the need for feminist and masculinities theories to understand them. Feminist theorists explore females’ experiences of gender inequality to understand the causes, and impacts, of female oppression, such as discrimination and violence against women (VAW) and focus on discovering ways to improve the social world for women (Van Gundy & Kappeler, 2014). Patriarchy, or male domination, is a primary factor that perpetuates VAW at a societal level (Policastro, 2015). Patriarchy, and its impacts, are experienced in the private and public sphere.

Focusing on intimate relationships, male VAW may result from several factors, including personal history, such as witnessing or experiencing DV in childhood, substance abuse, association with delinquent peers, and/or coercive controlling behaviors toward female partners (Johnson et al., 2019). Coercive control refers to the “micromanaging” of women by men because adherence to, and belief in, traditional male gender roles can result in males feeling entitled to control the way females behave (Stark, 2012, p. 207). When an intimate bond is shared, men have increased opportunity to assert control in the form of stalking, intimidation, and shaming, and it is often achieved through isolation or monitoring of a partner’s behavior (Stark, 2012).

Perpetrators of familicide often have histories of DV and, therefore, it can be argued that many familicides occur following a pattern of coercive control and are a final attempt by the male to control his intimate partner and child(ren). For example, when an intimate partner leaves the relationship, the male may feel his control has been lost. When the relationship has been characterized by coercive control, he may view killing his partner as the only way to regain the control to which he feels entitled. In such cases, he may also kill his children because his feelings of possessiveness and control extend to them (Daly & Wilson, 1988). According to masculinities theory, this need to be in control is a result of the social construction of masculinity.

According to Connell (1995), hegemonic (or dominant) masculinity outlines how males should behave in society and is associated with power, heterosexuality, and physical strength. According to this research, males are socialized to be aggressive, dominant, and in control; if they do not portray these characteristics, they are not masculine (Connell & Messerschmidt, 2005; Flood, 2018). In some cases, this results in males going to great lengths to reaffirm their status as men, some engaging in violence to do so. These males are often classified as engaging in toxic masculinity, which refers to stereotypical behavioral norms of masculinity which contributes to the oppression of females, males, and individuals who are gender diverse (Connell & Messerschmidt, 2005; Flood, 2018). Based on this literature, it can be argued that males who commit familicide engage in toxic masculinity and commit the killings to live up to or reaffirm their (and society’s) hegemonic masculine ideal.

Drawing from feminist and masculinities literature, familicide may occur when a male believes his masculinity has been threatened, particularly by a female partner whom he sees as his possession. Many individuals experience triggering events prior to committing familicide, which they may see as a threat to their control and/or cause shame (Websdale, 2010). According to feminist and masculinities theorists, males are socialized to think anger is an appropriate emotional response (Marganski, 2019; Websdale, 2010) and, by default, shame is not. As a result, some males attempt to demonstrate their masculinity by controlling those they see as inferior, as taught by patriarchal structures, such as their female partners and/or children. Therefore, men who kill their families may do so as a final attempt to conform to the standards of hegemonic masculinity, which positions men as superior to women and children in a patriarchal society.

## Methodology

### Data

Defining familicides as the killing of a current or former intimate partner and at least one biological or stepchild (Wilson et al., 1995), our research draws on data collected by the CDHPIVP, a project that explores factors related to DH generally and specific to four vulnerable populations^1^—Indigenous, immigrant/refugee, rural, remote, and northern (RRN^2^), and children exposed to DV. As part of this project, a quantitative national DH database was developed to document information collected from public documents (i.e., media files and court documents). This database includes information on victim, accused,^3^ and incident characteristics as well as criminal justice responses, agency involvement, and risk factors.^4^

Public documents were first screened to identify cases that met the CDHPIVP definition of DH.^5^ Once identified, all corresponding media files and court documents, where available, were collected and coded by a team of researchers. All documents were read, and information coded into a data spreadsheet. The coding was verified for accuracy by a senior research assistant, who also trained all coders, minimizing the need for inter-rater reliability checks.^6^ The database contains information on 677 DH cases, involving 770 victims, that occurred in Canada between and including 2010 to 2019. Of the 677 cases, 26 met Wilson et al.’s (1995) definitional criteria for familicide adopted in this study (i.e., the killing of a current or former intimate partner and at least one biological or stepchild), which involved 71 victims and 26 accused. These figures highlight the rarity of familicide relative to the total proportion of all domestic homicides. Specifically, during the 10-year time frame, less than 4% of all domestic homicides can be classified as familicide, comprising nine percent of all DH victims. As such, our data are based on a small number of cases, but their examination can provide context for these killings and contribute to enhanced risk assessment, risk management, and safety planning.

Just under two-thirds (65%) of all familicides (*N* = 17) involved the killing of a primary victim who belonged to one or more of the four vulnerable groups.^7^ Of the 71 total victims, 31 children (less than 18 years old) were killed alongside the primary victim in 21 cases. Among the 31 child victims, 14 lived in a RRN region, five were Indigenous, and two were second-generation immigrants, highlighting intersecting vulnerabilities and oppression. Another 17 adult victims, comprising 12 cases, were killed in a RRN area of the country, three of which were Indigenous. Five cases involved the killing of immigrants/refugees with a total of nine adult victims. Nine cases involved the killing of a primary victim who did not belong to any vulnerable group.

### Variables and Analyses

Victim and accused characteristics included gender, age, victim-accused relationship, and history of violence in the relationship. Incident characteristics included geographic and physical location of the familicide, cause of death, whether the accused attempted or died by suicide, and motivating factors. We conducted our analysis by exploring familicide case factors using univariate statistics to provide a general snapshot of the common characteristics of familicides that occur in Canada as well as by RRN populations. Almost half of all familicide victims (*N* = 31; 44%) belonged to RRN populations. Numbers were too small to focus meaningfully on the other three populations, although children are focused on due to our definition of familicide.

## Results

We present descriptive results in Table 1^8^ for victim, accused, and incident characteristics before discussing motivations for the killings.

**Table 1.** Descriptive Statistics: Victim, Accused, and Situational Variables of Familicide Cases in Canada, 2010 to 2019 (*N* = 26 cases; RRN *N* = 12 cases)^a^.

	Total sample		RRN
	*n*	%	*n*
*Victim and accused variables*			
Primary victim gender			
Female	25	96	12
Male	1	4	-
Secondary victim(s) gender			
Female	27	60	14
Male	18	40	5
Primary victim age			
18–24 years	3	12	1
25–34 years	10	38	5
35–44 years	7	27	1
45–54 years	5	19	4
55–64 years	1	4	1
Secondary victim(s) age			
Youth (0–17 years)	31	69	14
18–24 years	4	9	3
25–34 years	-	-	-
35–44 years	4	9	1
45–54 years	2	4	1
55–64 years	4	9	-
Accused gender			
Male	25	96	12
Female	1	4	-
Accused age			
18–24 years	2	8	1
25–34 years	10	40	3
35–44 years	4	16	2
45–54 years	8	32	4
55–64 years	1	4	1
Primary victim-accused relationship			
Legal partner	10	38	3
Common-law	6	23	5
Estranged partner	9	35	3
Boyfriend/girlfriend	1	4	1
Secondary relationship(s)			
Biological child of accused	20	44	9
Stepchildren of accused	13	29	8
Other kin of accused	2	4	1
Primary victim family	7	16	-
Other	3	7	1
*Situational variables*			
History of violence			
Physical violence	8		2
Emotional	5		3
Coercion/threats	3		1
Sexual	1		0
Economic	1		0
Other	1		1
Location of familicide			
Shared residence	46	65	20
Primary victim home	9	12	2
Accused’s home	5	7	2
Public street	5	7	3
Another residence	2	3	2
Other/unknown	4	6	2
Population density			
Rural	12	46	12
Urban	14	54	0
Cause of death			
Shooting	30	42	17
Stabbing	9	13	-
Strangulation	7	10	1
Beating	6	8	2
Vehicular death	3	4	3
Arson	2	3	-
Unreported	14	20	8
Perpetrator suicide			
No suicide	11	42	3
Attempted suicide	1	4	1
Died by suicide	14	54	8
Suicide method			
Shooting	8	57	5
Jump from height	2	15	-
Vehicular	1	7	1
Unknown method	3	21	2

a Figures above are primarily based on number of cases of familicide (*N* = 26), except for information relating to secondary victims, cause of death, and location of familicide which are based on the number of victims (*N* = 71). The same applies for familicide cases occurring in rural, remote, or northern areas (*N* = 12), where 31 total victims were killed. Percentages may not equal 100 due to rounding.

### Victim and Accused Characteristics

The univariate statistics indicate that, of the familicides committed in Canada between 2010 and 2019, primary victims in all but one case were adult females (*N* = 25; 96%) with one case involving an adult male victim.^9^ When looking at the overall victim sample (including primary and secondary victims), females comprised 73% (*N* = 52) of all victims. Focusing on primary victims specifically, the average age was 36 years with most (*N* = 10; 38%) being within the 25-to-34-year age group (see Table 1). Primary victims killed in a RRN area (*N* = 12) were slightly older, with an average age of 39 years. Beyond primary victims, 45 secondary victims were killed, including 27 females (60%) and 18 males (40%).^10^ When focusing on secondary victims specifically, the average age was 18 years, with the majority younger than 18 years (*N* = 31; 69%).

Most accused of familicide were male (*N* = 25; 96%) and one was female. Similar to the average age of primary victims, the accused had an average age of 39 years, with most falling within the 25-to-34-year age category (*N* = 10; 40%), followed closely by those aged 45-to-54 years (*N* = 8; 32%).^11^ Accused who committed familicide in RRN areas (*N* = 12) were slightly older with an average age of 42 years. Although not shown in Table 1, based on information available (62% of cases), just over one-third of accused were employed (*N* = 9; 35%) and seven were unemployed or not working at the time of the familicide (27%).

#### Victim and accused relationship

Of the 26 familicides, the primary victim was either the legal partner of the accused (*N* = 10; 38%) or an estranged legal or common-law partner (*N* = 9; 35%), followed by current common-law partners (*N* = 6; 23%), and one current girlfriend (4%). Among those who lived in RRN areas (*N* = 12), most were in common-law relationships (*N* = 5; 42%), followed by legal marriages (*N* = 3; 25%), estranged marital relationships (*N* = 3; 25%), and one dating relationship (8%).

In addition to physical separation, another 20% of cases involved those who were still together, but there was evidence that separation was imminent (i.e., the victim had told the accused she was leaving, arranged alternative accommodations, and/or expressed her desire to leave the family) (*N* = 5). Most secondary victims were the biological children of the accused and primary victim (*N* = 20; 44%), followed by stepchildren of the accused (*N* = 13; 29%). The remaining 12 victims shared varying relationship types; most were family members of the primary victim (*N* = 7; 16%) or the accused (*N* = 2; 4%). Others were the primary victim’s new partner (*N* = 1; 2%), or friend (*N* = 1; 2%) and one was a stranger to the accused (2%).^12^ Among secondary victims killed in RRN areas (*N* = 19), most were the accused’s biological child (*N* = 9; 47%) or stepchild (*N* = 8; 42%), with the remaining two being the accused’s parent or primary victim’s new partner.

#### Prior history of violence

There was a documented history of violence by the accused against the primary victim in half of all cases (*N* = 13; 50%). Most often prior violence was physical (*N* = 8) followed by emotional/psychological abuse (*N* = 5), coercion/threats (*N* = 3), and an equal proportion of sexual, economic, or another type of violence (*N* = 1 each).^13^ Victims were most likely to report violence to police (*N* = 4), family members (*N* = 4), or both (*N* = 1), with the remaining confiding in friends (*N* = 2), coworkers (*N* = 1), or neighbors (*N* = 1). In five RRN cases (42%) there was a documented history of violence; however, contrary to the patterns above, none reported the violence to police, instead confiding in family (*N* = 4) or neighbors (*N* = 1). It is expected that these numbers are an underestimation of the history of DV in these cases.

### Incident Characteristics

Most victims were killed in a residence they shared with the accused (*N* = 46; 65%). This was also the case for familicides in RRN areas (*N* = 12), where two-thirds occurred in a shared residence (*N* = 8). The next largest group of victims were killed in their residence not shared with the accused (*N* = 9; 12%), the accused’s home (*N* = 5; 7%), or a public street (*N* = 5; 7%). The remaining victims were killed in another residence (*N* = 2; 3%) or other/unknown locations (*N* = 4; 6%).^14^ When focusing on population density, almost an equal proportion occurred in rural (*N* = 12; 46%) and urban (*N* = 14; 54%) areas.^15^

The cause of death was not always reported in public documents (information was missing for 20% of victims). When this information was known, most victims were shot to death (*N* = 30; 42%). This finding is driven largely by RRN cases, where nearly three-quarters of victims were shot (*N* = 17; 74%). An equal number of victims were killed with a handgun or long gun (*N* = 10 each). The type of gun used was not available for eight victims. In the total sample of familicides, the second largest group of victims were stabbed (*N* = 9; 13%), followed by strangulation (*N* = 7; 10%), beating (*N* = 6; 8%), vehicular deaths (*N* = 3; 4%), and arson (*N* = 2; 3%).

Over half of all perpetrators (*N* = 14) died by suicide following the familicide and one attempted suicide. Most perpetrators died immediately after (*N* = 5) or within a day (*N* = 5) of the familicide. Among the perpetrators who died, the largest group shot themselves (*N* = 8; 57%), followed by those who jumped from a height (*N* = 2; 15%), and one intentionally drove his vehicle into oncoming traffic (7%). The remaining three killed themselves using unknown methods. There was evidence that suicide was part of the original homicide plan in seven cases (44%) (e.g., suicide note left behind), but information for the remaining familicides was not available. These figures are driven largely by RRN cases (*N* = 12), where eight perpetrators (67%) died by suicide.

### Motivating Factors

Among the 26 cases, 13 involved a triggering event that may have motivated the familicide. In four cases, the primary victim and accused were in the process of separation or divorce. One accused in this group was laid off on the same day the separation papers arrived which meant he faced losing the family home and, potentially, custody of their children. Another case involved an accused who found out 3 days before the familicide that the primary victim (his estranged female partner) had a new partner. Aside from separation as a triggering event, one accused filed for bankruptcy, two were reportedly struggling with mental health issues following job losses, one was experiencing desperation over a failed business, associated financial frustrations, and a looming deportation order, and another was under investigation for serious allegations concerning children. One accused was increasingly stressed over his daughter’s constant migraines and killed her, his wife, and his sister. The final cases involved an accused who had been released from incarceration the week prior and an accused who signed a probation order one week prior to killing his estranged wife and two children, agreeing to not contact them.

## Discussion and Conclusion

The purpose of our study was to gain a better understanding of what familicide looks like in Canada. In support of our hypotheses, and in line with prior literature, the results showed that familicides in Canada were gendered, with accused being primarily male and victims primarily female (Karlsson, Malén, et al., 2021; Mailloux, 2014). This section highlights the contributions of our study prior to discussing limitations and directions for future research. Due to the rarity of familicide in Canada, prediction is not possible; therefore, it remains difficult to use common characteristics for prevention efforts given they are common in other types of DH as well. Our recommendations highlight avenues for further investigation in future studies with larger sample sizes to build upon our findings, which provide a crucial starting point in the Canadian context.

In support of Hypothesis 1 and prior research, one half of the accused in our sample had a history of DV (Dobash et al., 2007; Johnson & Sachmann, 2014; Taylor, 2018), which highlights DV as a risk factor for familicide. This finding supports the need for a gendered framework when analyzing familicide, as some result from an escalation of DV which appear to occur when males experience triggering events connected to their female partner, which supports Hypothesis 2. This finding demonstrates that DV can take many forms, as the victims experienced not only physical violence but also emotional/psychological abuse and coercion/threats, thus, emphasizing the need for practitioners and frontline workers to work to identify other types of DV beyond physical violence. The purpose of our study was not to test feminist and masculinities theories but rather to analyze one type of male VAW (i.e., familicide) through this theoretical lens. Our findings appear consistent with these theories and support recent literature that has recognized the risks associated with coercive control (Johnson et al., 2019; Stark, 2012). As such, our study demonstrates the continuing importance of further research drawing from both feminist and masculinities theories to better understand cases of familicide and VAW more generally.

Consistent with prior literature, and in support of Hypothesis 3, firearms were the most common weapon used in our sample, making firearm ownership a substantial risk factor for familicide and a crucial area for reform (Fegadel & Heide, 2017; Karlsson, Antfolk, et al., 2021). According to a recent US study, strict firearm legislation that took histories of DV into consideration was associated with lower levels of IPH (Zeoli et al., 2018). However, the implementation of firearms licensing in 2001 and the firearms registry in 2003 did not affect rates of homicide in Canada (Langmann, 2012). As such, we recommend that laws are strengthened to make legal firearm ownership more difficult, especially in rural areas where long-gun ownership is common and where familicide occurs most frequently. In doing so, it may reduce the risk of familicide. Although this recommendation is based on a small number of familicides, literature on DV and DH demonstrates the importance of minimizing firearm ownership in rural areas (Dawson et al., 2021; Doherty & Hornosty, 2008) and our findings support these recommendations based on one type of DH that has received limited attention. In Canada, current firearm legislation requires a chief firearms officer or provincial court judge to be informed if an individual has used, or threatened, violence toward their current/former intimate partner to determine whether they are eligible to hold a firearms license (*Firearms Act*, 2019). Based on our findings, we argue that this legislation has not prevented individuals who have histories of DV from legally acquiring and owning firearms. Future research is needed to understand why this occurs (i.e., judicial discretion).

Although controversial in most countries, research provides support for the above recommendations, as studies show decreases in IPH in U.S. states where policies restricted individuals with DV restraining orders from accessing firearms (Vigdor & Mercy, 2006; Zeoli & Webster, 2010). Other revisions could include further screening, improved psychiatric testing, and greater difficulty (e.g., course requirements, increase in price) to acquire a firearm, particularly in rural areas. A recent review of literature analyzing the effect of firearm restriction laws on homicides internationally found that such policies are associated with lower rates of firearm related deaths, including IPH, in several countries including Canada, Australia, and New Zealand (Santaella-Tenorio et al., 2016). As such, although this recommendation is based on a small sample, our study provides support for other multi-country studies that recommend legislations that decrease firearms misuse.

In our study, most perpetrators died by suicide following the familicide, which supported Hypothesis 4 and prior literature (Liem & Koenraadt, 2008; Liem & Reichelmann, 2014; Mailloux, 2014; Taylor, 2018). Our findings indicate that suicidal ideation may be a risk factor for familicide, particularly when perpetrators have histories of DV. This finding emphasizes the importance of raising awareness among mental health professionals about the need to assess DV among men with suicidal ideation for risk of familicide and other types of DH. The importance of increased awareness is supported by Banks et al. (2008), who suggest that identification and treatment of suicidal ideation may prevent suicide and IPH followed by suicide. Our findings also emphasize the need for risk management strategies geared toward individuals who have histories of DV and have expressed suicidal ideation or previous attempts. Moreover, the fact that most suicides involved a firearm further emphasizes the need for an amendment to current firearm legislation. As a result, it may be possible to prevent suicide from occurring and/or from escalating to eventual homicide-suicide, including familicide (Johnson & Sachmann, 2014).

Familicides occur more frequently in rural, rather than urban, areas (Duwe, 2004; Gallup-Black, 2005). Our study found an almost equal proportion of familicides in each area; however, nearly half of the victims in our sample belonged to a RRN population, despite research showing that only 16% of the Canadian population lives in rural areas (Beattie et al., 2018). Therefore, our findings support Hypothesis 5 and highlight the need for prevention measures specific to these locations, such as paying more attention to risk at the community level and exploring how various social identities intersect to increase risk of familicide in these areas (Straatman et al., 2019).

Drawing from our theoretical framework, our findings emphasize the importance of integrating material into rural and urban school curricula that educate children and adolescents on the issue of VAW and gender inequality. It would also be beneficial to incorporate prevention measures, such as promoting emotional regulation, to better address adverse childhood experiences. Doing so may reduce the desire to achieve a hegemonic masculine ideal, which may help end the cycle of women and children being viewed in proprietary terms and reduce their risk of victimization and, thus, familicide. Our findings also highlight the importance of analyzing intersecting risk factors, such as accused’s who have histories of DV, access to firearms, and reside in rural areas, and exploring how such circumstances may increase the risk of familicide and DH. Analyzing the intersection of risk factors is crucial, as research shows that clusters of risk factors increase the overall risk of DH occurring (Dawson & Piscitelli, 2021).

Our data and conclusions are based on the information available in public documents which is a limitation. A substantial proportion of cases were missing data for many variables, which restricted us from analyzing what role the accused’s mental health played in the familicide, for example. It may also be that more domestic homicides occurred but were not labeled as such publicly, resulting in an undercount of our sampling population. Likewise, other variables reported in this study, such as histories of DV, underestimate the true prevalence experienced by the victim due to underreporting (Fernández-Fontelo et al., 2019). Further, it was not possible to analyze differences in racial and ethnic trends with regard to primary victims and accused, nor how such trends compare to homicide generally.^16^ These limitations are similar to other studies that examine homicide, largely stemming from the data sources available; however, some research has shown that media are often as reliable as official data for some variables (Parkin & Gruenewald, 2017). Despite these limitations, our study provides a first look at the characteristics of familicide in Canada, including key risk factors, highlighting directions for future research.

For instance, our findings highlight the importance of developing a secure and comprehensive DH database across Canada, particularly for cases where public records are limited (i.e., homicide-suicide)—an implication that is applicable globally (Cullen et al., 2021). Currently, two data sources capture homicide in Canada: The Homicide Survey and the Mortality Database. These sources have reliability issues, as the Homicide Survey generates higher homicide counts than the Mortality Database and the two are not consistent in terms of homicide trends (Gabor et al., 2003). Moreover, homicide data often have issues with validity, can be difficult to access, rarely focus on justice and accountability, overlook killings that are enabled by the state (e.g., deaths that occur in custody), and fail to consistently measure sex/gender related motivations and indicators (Dawson & Carrigan, 2021). A comprehensive database should compile information from medical examiners, coroners, police services, and other organizations such as social service agencies, where possible, and be available to researchers and policy makers. Due to large amounts of missing information, particularly in DH cases, there should be a standard and comprehensive data form completed by all actors listed above to better inform these efforts. As a result, researchers and policy makers will not be obliged to rely on media and court decisions alone.

The fact that our study involved 26 cases of familicide and 71 victims also emphasizes the importance of classifying certain familicides as mass homicides. To illustrate, 46% (*N* = 12) of all familicides in our study involved three or more victims. Of these 12 cases, 43 victims were killed. Mass homicides generate an enormous amount of attention, particularly from the media; however, most cases that garner media coverage do not involve a familial accused (Duwe, 2004; Fridel, 2017) and, consequently, research focuses on non-familial mass homicides. In reality, most mass homicides that occur in Canada are familial, as they typically involve males who kill multiple members of their family (Boyd, 2021; Duwe, 2004). By recognizing familicide as a distinct homicide subtype, it highlights the importance of classifying familicides that involve three or more victims as familial mass homicides. As a result, researchers will gain a better understanding of familial homicides, which may aid in the development of risk assessment strategies.

Our findings demonstrate that familicides in Canada are similar to other countries; however, they reveal the benefits of exploring familicide using a gendered theoretical framework. Specifically, certain familicides can likely be understood as a result of the social construction of masculinity in a patriarchal society that positions hegemonic masculinity as the norm, resulting in women and children being viewed as inferior to men. Our findings draw attention to several risk factors for familicide and, thus, highlight the need for risk assessment, risk management, and safety planning strategies to address warning signs for familicide which may differ from those relevant to other domestic homicides (Douglas & Kropp, 2002). Although our study is based on a small sample size, our findings provide a first look at familicide in Canada and demonstrate the importance of taking an intersectional approach when exploring familicide and other forms of gender-based violence. Specifically, doing so will highlight the increased vulnerability of certain populations and aid in the identification of prevention measures for these groups (Etherington & Baker, 2018). As a result, it may be possible to develop interventions that can effectively minimize the risk of familicide, and other domestic homicides, occurring in the future.

## Notes

1. These four populations are defined as vulnerable by the CDHPIVP due to their increased risk of domestic homicide compared to other populations more generally.

2. RRN refers to small towns with fewer than 10,000 residents as well as remote and Northern populations.

3. We use the word “accused,” as opposed to “perpetrator,” because some cases have not yet been fully processed through the courts. However, when discussing individuals who died by suicide following the familicide, the word “perpetrator” is used because the individual will never legally be accused of anything as they are dead but have been identified as the perpetrator.

4. For more information on the database, see www.cdhpi.ca.

5. The CDHPIVP defines domestic homicide as the killing of a current or former intimate partner, their child(ren), and/or other third parties. An intimate partner can include people who are in a current or former married, common-law, or dating relationship. Other third parties can include new partners, other family members, neighbors, friends, coworkers, helping professionals, bystanders, and others killed as a result of the incident (see www.cdhpi.ca).

6. Data were trusted to be accurate; however, if data sources conflicted, information reported in court documents was deemed more reliable than what was reported in media coverage.

7. Primary victim refers to the intended target, or the individual who shares the closest relationship to the accused and is, therefore, predominantly an intimate partner.

8. The table is not broken down by secondary victims, as important characteristics are noted, and the remainder of the data was not unique to primary or secondary victims.

9. Each case has one primary victim and, therefore, the terms “primary victims” and “cases” can be used interchangeably.

10. When focusing on child victims only, females comprise just over half of all secondary victims (55%; *N* = 17), with 14 male child victims (45%). Females also comprise over two-thirds (71%; *N* = 10) of all adult secondary victims with four adult male victims (29%).

11. The age of one accused was unknown.

12. The numbers do not add up perfectly here due to rounding.

13. Five cases involved multiple types of reported violence, which explains the values exceeding 13. The “other” type of violence involved the accused locking his partner inside their home when he left the residence.

14. The exact location of homicide was unknown for two familicide victims because their bodies were found in a wooded area. The remaining two victims were killed in a vacation residence/chalet that one victim owned but was not her primary residence.

15. Rural is conceptualized as having a population of less than 10,000 inhabitants, whereas urban includes all areas with a population greater than 10,000 inhabitants.

16. There has been a long-standing, oftentimes informal, ban on the collection and dissemination of data (primarily for researchers) that identifies race/ethnicity of victims and perpetrators of crime (Dawson, 2017), except for Indigenous identity by Statistics Canada. There have been recent calls for more race-based data collection in Canada, but the past situation has limited the ability to focus on race/ethnicity as an explanatory variable in Canadian studies.
